# Dynamics of bacterial and fungal communities associated with eggshells during incubation

**DOI:** 10.1002/ece3.1011

**Published:** 2014-03-08

**Authors:** Stéphanie Grizard, Francisco Dini-Andreote, B Irene Tieleman, Joana F Salles

**Affiliations:** 1 Department of Animal Ecology, Centre for Ecological and Evolutionary Studies, University of GroningenNijenborgh 7, Groningen, NL-9747 AG, The Netherlands; 2 Department of Microbial Ecology, Centre for Ecological and Evolutionary Studies, University of GroningenNijenborgh 7, Groningen, NL-9747 AG, The Netherlands

**Keywords:** Birds, eggshells, incubation, microbes, molecular tools

## Abstract

Microorganisms are closely associated with eggs and may play a determinant role in embryo survival. Yet, the majority of studies focusing on this association relied on culture-based methodology, eventually leading to a skewed assessment of microbial communities. By targeting the 16S rRNA gene and internal transcribed spacer (ITS) region, we, respectively, described bacterial and fungal communities on eggshells of the homing pigeon *Columba livia*. We explored their structure, abundance, and composition. Firstly, we showed that sampling technique affected the outcome of the results. While broadly used, the egg swabbing procedure led to a lower DNA extraction efficiency and provided different profiles of bacterial communities than those based on crushed eggshell pieces. Secondly, we observed shifts in bacterial and fungal communities during incubation. At late incubation, bacterial communities showed a reduction in diversity, while their abundance increased, possibly due to the competitive advantage of some species. When compared to their bacterial counterparts, fungal communities also decreased in diversity at late incubation. In that case, however, the decline was associated with a diminution of their overall abundance. Conclusively, our results showed that although incubation might inhibit microbial growth when compared to unincubated eggs, we observed the selective growth of specific bacterial species during incubation. Moreover, we showed that fungi are a substantial component of the microbial communities associated with eggshells and require further investigations in avian ecology. Identifying the functional roles of these microorganisms is likely to provide news insights into the evolutionary strategies that control embryo survival.

We aimed to describe the dynamics of bacterial and fungal communities on homing pigeon eggshell surfaces. We investigated these communities at early and late incubation stages.

## Introduction

Microorganisms in close interaction with eggs may act as a selective force on avian hatching success (Cook et al. 2003, 2005a; Beissinger et al. 2005). In this earliest stage of life, they may be harmful because of their potential pathogenicity against embryos. However, only a small subset of bacterial species might be actually pathogenic to the embryo. Thus, an increase in the number of nonpathogenic bacteria during incubation could be seen as an complementary parental approach to avoid colonization by pathogenic ones though direct inhibition or competitive exclusion (Cook et al. 2005b). Understanding which factors drive microbial communities on eggshells may lead to a better comprehension of evolutionary strategies that improve embryo survival. Environmental components, parental physiology and behavior, and their interactions, are key drivers of these microbial communities (Ruiz-de-Castañeda et al. 2011a). Environmental components such as protection against adverse conditions, nest structure, reuse of a nest, and choice of lining materials (e.g., feathers) can influence bacterial loads on eggshells (Baggott and Graeme-Cook 2002; Peralta-Sanchez et al. 2010; Walls et al. 2012). For instance, eggs from nest boxes exhibited lower bacterial and fungal growth than eggs from open-cup nests (Godard et al. 2007). Parental physiology affects eggshell microbiota through vertical transmission of cloacal microflora (Ruiz-de-Castañeda et al. 2011b; Ruiz-De-Castañeda et al. 2011c), which has been implicated as a source of bacterial inoculation during egg-laying (Barrow 1994). Finally, parental incubation behavior has been found to either reduce or limit bacterial growth on the eggshell surface (Cook et al. 2005b; Shawkey et al. 2009; D'Alba et al. 2010) or to decrease bacterial and fungal invasion of egg contents by limiting trans-shell infection (Cook et al. 2003, 2005a), when compared to eggs that are left exposed (unincubated).

The danger that microbes on eggshells present to the embryo stems from their ability to invade egg contents through the pores in the eggshell, inducing hatching failure (Cook et al. 2003, 2005a). Parental incubation behavior was shown to limit bacterial growth by maintaining shell dryness (D'Alba et al. 2010; Ruiz-De-Castañeda et al. 2011c) and by controlling bacterial richness (Shawkey et al. 2009). An early onset of incubation in the Pied Flycatcher *Ficedula hypoleuca* led to bacterial growth inhibition (Ruiz-de-castañeda et al. 2012; but see Walls et al. 2012). In addition, experimentally exposed eggs under tropical conditions (Cook et al. 2003, 2005a) and artificially wetted eggs (D'Alba et al. 2010) had higher bacterial loads, as determined by plate counts, than dry ones. Interestingly, this effect of incubation was not replicated in eggs from a temperate region (Wang et al. 2011). Although incubation controls for bacterial richness and abundance, as compared to exposed eggs, little is known about the changes in microbial communities during incubation. Comparisons between early and late incubated eggs have suggested that incubation does not lead to changes in bacterial community structure (Shawkey et al. 2009; Ruiz-de-Castañeda et al. 2011b). However, due to the small number of studies and limitations associated with methodological issues, these results cannot be generalized to other bird species.

Additionally, while the majority of studies focused on the bacterial domain, fungi have been poorly described during egg development, despite their presence in the nest environment (Baggott and Graeme-Cook 2002; Goodenough and Stallwood 2010, 2012), in the adult bird plumage (Camin et al. 1998; Mandeel et al. 2011), and on poultry egg surfaces (Szablewski et al. 2010; Nowaczewski et al. 2011). Fungi might play an active role in microbial invasion as their hyphae can penetrate the eggshell leading to an increase in the number of unplugged pores, which can be used by pathogenic bacteria as a direct route to egg contents (Board and Tranter 1995). Only a few studies have investigated fungal presence/absence on eggshells (Cook et al. 2005a,b2005b; Godard et al. 2007), and only a single one has attempted a classification to the genus level based on mycelium characteristics (Cook et al. 2003).

These pioneering studies on eggshell microbiota have provided the first steps in identifying egg-related microorganisms, which is of primary importance to fully understand their roles. Yet, the majority of them have relied on culture-based methodology, where bacterial groups are the main focus, and are usually assessed by counting colony-forming units (CFUs) in semi-selective media. However, the “great plate count anomaly” states that only 0.1–10% of all microbes can be cultured under laboratory conditions (Staley and Konopka 1985; Amann et al. 1995; Hugenholtz 2002) indicating that plating techniques provide a skewed assessment of microbial communities. For instance, it has recently been shown that culture-dependent (plating) and independent (molecular) methods revealed different bacterial communities associated with bird feathers (Shawkey et al. 2005) and, more specifically, differences in *E. coli* abundance on eggshells (Lee et al. 2013). Studies using molecular techniques (PCR-TGGE) to assess egg-related microbes showed that bacterial communities differed within and between clutches of six avian species (Martín-Platero et al. 2010). In addition, the sequencing of a subset of bacterial colonies (Wang et al. 2011) and the use of PhyloChip microarrays (Shawkey et al. 2009) characterized up to thirty bacterial genera on eggshells of three bird species, and almost 1500 unique bacterial taxa on the eggshells of Pearly-eyed Thrashers *Magarops fuscatus*, respectively.

The description of microbial communities associated with eggs using molecular tools can be a challenge, especially when the study design limits the choice of sampling strategies. In most cases, when eggs need to hatch for further study, eggshell swabbing is the only possibility, and this nondestructive sampling is still broadly used to collect bacterial cells. However, some cells most likely escape this technique, because eggshells are of complex structure, including diverse calcified layers (Karlsson and Lilja 2008), variable thickness, and the presence of pores (Massaro et al. 2004; Zimmermann and Hipfner 2007), which provide potential hideouts for microorganisms [e.g., in the poultry industry, *Salmonella* cells have been recovered from eggshell pieces, after rinsing the egg surface (Kawasaki et al. 2008)].

The aim of this work was to describe the microbial communities associated with eggshells at early and late incubation using complementary molecular tools. Before doing so, we compared two different sampling protocols: a noninvasive egg swabbing and a destructive crushing based on eggshell pieces, to investigate whether they yielded different microbial communities. We used the eggshell pieces approach, which targets the microbial cells living on the eggshell and in the eggshell pores, to describe both bacterial and fungal communities of the eggs of homing pigeons *Columba livia*. Specifically, we characterized the structure, abundance, and composition of these communities, using denaturing gradient gel electrophoresis (PCR-DGGE), quantitative PCR (qPCR), and DNA sequencing, respectively. By targeting both microbial groups to the same extent, we aimed at characterizing the fungi on avian eggshells as well as improving our knowledge on bacterial-egg association. Considering that incubation selectively influences microbial communities, we predicted that bacterial and fungal communities will change between early and late incubated eggs, leading to a rearrangement in the community structure as well as a lower bacterial and fungal abundance and diversity at the end of incubation.

## Materials and Methods

### Egg swab and eggshell collection

From May to July 2011, we collected 28 eggs from six female pigeons (*Columba livia)* living in the outdoor aviaries of the University of Groningen (53°13′N, 6°33′E, the Netherlands). We housed females separately from males. Females exhibited incubation behavior, despite their eggs being unfertilized. We collected and handled samples wearing gloves sterilized with 70% ethanol. Samples were individually stored in sterile containers and frozen at −20°C until processing.

First, to compare eggshell swabbing and eggshell crushing methods, we made swabs and sampled eggshell pieces (*n *= 10 eggs). We sampled each of these eggs using both methods. For swabs, we rubbed one-fifth of the total eggshell surface using ClassiqSwabs (Copan Flock Technologies, Brescia, Italy), slightly wetted in a sterile solution (phosphate-buffered saline [PBS]/0.05% Tween-80). We marked swabbed areas with a pencil to avoid sampling the same section more than once. To sample eggshell pieces, we used frozen eggs and removed the eggshell from the albumen with a sterilized spatula. We ensured that the mass of eggshell collected for the crush method corresponded to the area of eggshell surface swabbed with the swab method (range: 0.36–0.48 g).

Afterward, to perform cross-comparison between microbial communities of early and late incubated eggs, we collected eggs within 24 h after laying and after an incubation period of seventeen to eighteen days, corresponding to the last day of incubation if eggs were fertilized. We dissected eggs as previously mentioned and worked with eggshell pieces instead of egg swabs.

### DNA extraction protocols from egg swabs and eggshells

To evaluate the efficiency among different methods to extract total DNA from bird eggs, we applied four different protocols for egg swabs and two different protocols for eggshells. For the four egg swab methods, we first harvested bacterial cells following Martín-Platero et al. (2010), slightly modified. We added 700 *μ*L of PBS/0.05% Tween-80 to each tube containing a swab. We used an extra 0.2 mL PCR tube to prevent swabs from going through the 0.5 mL tube. This pellet was then used in one of the four different protocols chosen based on previous studies. These protocols were as follows: (i) Chelex-100 (chelex) (Martín-Platero et al. 2010), (ii) DNeasy® Tissue kit (DNt; Qiagen, Valencia, CA) supplemented with Gram-positive bacteria (Shawkey et al. 2009), (iii) NucleoSpin® Tissue kit (NSt; Macherey-Nagel, Leiden, the Netherlands) for bacteria, based on chemical cell disruption, and (iv) Fast DNA® SPIN kit for soil (FSs; MP Biomedicals LLC, Solon, OH) based on mechanical cell disruption. Kits were used according to their manufacturer's instructions. Additionally, each of these four protocols was run once with a sterile swab to verify their sterility, and the resulting extraction was used as template in PCR as “DNA blank” controls (see sections on bacterial and fungal PCRs).

To explore the two DNA extraction protocols for eggshells, we crushed eggshell pieces in liquid nitrogen using mortar and pestle previously washed and sterilized by autoclaving for 30 min at 120°C. Each mortar and pestle was used only once and then washed and autoclaved again. We extracted DNA using the NucleoSpin® Soil kit (NSs; Machery-Nagel) and the aforementioned FSs kit (MP Biomedicals), following manufacturer's instructions, except that the cell disruption step was achieved by bead beating. Tubes containing eggshell powder were placed in a Mini-Beadbeater (BioSpec Products Inc., Bartlesville, OK) and processed either two times for 60 s (NSs) or one time for 40 s (FSs). The NSs kit was run with SL2 buffer (see kit instructions for details). In brief, NSs kit came along with two lysis buffers (SL1 and SL2) and an enhancer SX, to test four different lytic procedures: SL1 buffer alone, SL1 buffer with SX, SL2 buffer alone, and SL2 buffer with SX. After testing all possibilities, we opted for SL2 buffer alone as it provided highest DNA yield. We also ran each of these protocols with their respective solutions as template on “DNA blank” controls in later PCRs (see sections on bacterial and fungal PCRs). For both kits, DNA was eluted twice per sample, with a final volume of 200 *μ*L.

### DNA quantification by fluorescence

We used the Quant-iT™ PicoGreen® dsDNA kit (Molecular Probes Inc., Eugene, OR) to quantify DNA, following the manufacturer's recommendations. Samples were run in 96-well flat-bottom black microtiter plates (Nunc 165305; Thermo Scientific, Rochester, NY). A nine-point standard curve was achieved using Lambda phage DNA, from 1500 ng/mL to 1 ng/ml. Each plate reading was carried out with a standard curve, and all samples were run in triplicate. Wells were filled with either 80 *μ*L Lambda phage DNA (standard curve) or 15 *μ*L sample DNA completed with 65 *μ*L of PicoGreen working solution (see kit instructions). Readings were carried out with an Infinite® 200 Pro (Tecan Benelux, Giessen, the Netherlands), preceded by 3 s shaking and 10 s waiting.

### Bacterial PCR for DGGE

PCR amplifications targeted the 16S rRNA gene for bacteria, using a nested approach. The first run was carried out using B8F (5′-AGAGTTTGATCMTGGCTCAG) and 1492R (5′-GGTTACCTTGTTACGACTT) primers (Heijs et al. 2007; Raji et al. 2008). Each PCR was carried out in 25 *μ*L. It contained 19 *μ*L of PCR mix containing: 0.20 mmol/L of dNTPs, 0.25 *μ*mol/L of each primer, 0.625U TaqDNA polymerase (GoTaq® DNA polymerase; Promega, Madison, WI), 1.0 mg/mL BSA, 1× PCR buffer and nuclease-free water, and 6 *μ*L of DNA template (100 ng/mL). The amplification was carried out by performing an initial denaturation step (94°C for 5 min), followed by 20 cycles at 94°C for 1 min, 55°C for 1 min, and 72°C for 2 min, and finalized by an extension step at 72°C for 10 min. The second run (nested PCR) was performed using the primer pair F968-GC (5′-**CGCCCGGGGCGCGCCCCGGGCGGGGCGGGGGCACGGGGGG**AACGCGAAGAACCTTAC) and R1401-1b (5′-CGGTGTGTACAAGACCCGGGAACG) (Heuer et al. 1997; Brons and van Elsas 2008). The nested PCR was carried out in a 50-*μ*L reaction containing 2 *μ*L of the first PCR as template, using the same PCR mix described above, except that 1.25U TaqDNA polymerase was used. Amplification was performed following a touchdown approach. After one step denaturation at 94°C for 5 min, the touchdown step started, consisting of 10 cycles of denaturation at 94°C for 1 min, 1 min annealing at 60–55°C, and extension at 72°C for 2 min. Annealing temperature started at 60°C and decreased of 0.5°C every cycle until 55°C. The touchdown step was followed by additional 25 cycles at 94°C for 1 min, 55°C for 1 min, and 72°C for 2 min and finalized with an extension step at 72°C for 10 min. To verify the lack of contamination in the PCR, all PCRs contained a negative control comprising UltraPure™ DEPC-Treated Water (Invitrogen, Carlsbad, CA) and a second negative “DNA blank” controls to verify the sterility of the extraction procedures. None of them showed amplification. All PCRs were run in a Veriti® 96-Well Thermal Cycler (Applied Biosystems, Foster City, CA). PCR product concentrations were assessed within a 1.5% (w/v) agarose-TAE gel (95V), staining for 15 min in ethidium bromide. We compared the integrity, quantity, and size of the amplification products with a molecular weight marker (Smart Ladder; Eurogentec, Seraing, Belgium).

### Fungal PCR for DGGE

Fungal communities were assessed by amplification of the internal transcribed spacer (ITS) region for fungi, using a nested approach. The first run was performed using EF4 (5′-GGAAGGGRTGTATTTATTAG) and ITS4 (5′-TCCTCCGCTTATTGATATGC) primers (White et al. 1990; Smit et al. 1999). Each PCR was carried out in 25 *μ*L. It contained 19 *μ*L of PCR mix containing: 0.20 mmol/L of dNTPs, 0.25 *μ*mol/L of each primer, 2.5U TaqDNA polymerase (BIOTAQ™; Bioline, London, UK), 2 mmol/L MgCl2, 1 mg/mL T4 gene 32 protein (New England BioLabs Inc., Beverly, MA), 1× PCR buffer, and nuclease-free water and then completed with 6 *μ*L of DNA template (100 ng/mL). The amplification was carried out by performing an initial denaturation step at 94°C for 5 min, followed by 34 cycles at 94°C for 30 s, 55°C for 30 s, 72°C for 90 s, and finalized by an extension step at 72°C for 5 min. The second run (nested PCR) was carried out using the primers ITS1-F containing the GC-clamp (5′-**CGCCCGCCGCGCGCGGCGGGCGGGGCGGGGGCACGGGGGG**CTTGGTCATTTAGACTTGGTCATTTAGA) and ITS-2 (5′-GCTGCGTTCTTCATCGATGC) (White et al. 1990; Gardes and Bruns 1993). The nested PCR was carried out with a 50-*μ*L reaction containing 2 *μ*L of the first PCR as template. The PCR mix was the same as for the first run, except that T4 gene 32 protein was not added, and 1.25U TaqDNA polymerase was used. The amplification started by an initial denaturation step at 94°C for 5 min, followed by 34 cycles at 94°C for 30 s, 55°C for 30 s, 72°C for 30 s, and finalized by an extension step at 72°C for 5 min. Similarly to bacterial PCRs, all PCRs were run with negative controls of UltraPure™ DEPC-Treated Water (Invitrogen) and with “DNA blank” negative controls. None of them showed amplification. For PCR equipment and verification of PCR products, see bacterial PCR for DGGE section.

### DGGE community fingerprinting

We generated DGGE profiles using an Ingeny Phor-U® system (Ingeny International, Goes, the Netherlands). Amplicons (150 ng per lane) were loaded onto 6% (w/v) polyacrylamide gels in 0.5× Tris-acetate-EDTA (TAE) buffer, and we added the same reference marker to each gel for normalization purpose in computer analyses. We optimized our own marker for bacteria and used a 1 kb DNA ladder (O'Gene Ruler; Thermo Scientific, Vilnius, Lithuania) for fungi. Amplicons were run either in a 40–55% (bacteria) or in a 20–55% denaturant gradient (fungi), at 60°C for 16 h and at a constant voltage of 100V. After the run, gels were stained with SYBR gold (Molecular Probes, Leiden, the Netherlands) in 0.5× TAE buffer, in the dark for one hour. Then, we digitized DGGE profiles using a digital camera and stored as them TIFF files for computer analysis. Gels were normalized using the GELCOMPAR II software (Applied Maths, Sint-Martens-Latem, Belgium). After normalization, we compared community compositions by clustering lanes by Pearson's correlation coefficient implemented in the GELCOMPAR II software using the unweighted-pair group method with arithmetic mean, rolling-disk background subtraction, and no optimization (Rademaker et al. 1999; Kropf 2004).

### Quantification of bacterial and fungal abundance by quantitative PCR (qPCR)

Bacterial abundance was determined by quantifying 16S rRNA gene copy numbers using FP16S (5′-GGTAGTCYA YGCMSTAAACG-3′) and RP16S (5′-GACARCCATGCA SCACCTG-3′) (Bach et al. 2002). PCR mix consisted of 0.3 *μ*mol/L of each primer, 0.5× Power SYBR® Green (PCR Master Mix; Applied Biosystems, Paisley, U.K.), completed with nuclease-free water. Each PCR contained 23 *μ*L of PCR mix and 2 *μ*L of DNA template (100 ng/mL). The PCR program consisted of an initial denaturation step at 95°C for 10 min, followed by 40 cycles at 95°C for 20 s, 62°C for 60 s, and 72°C for 30 s.

Fungal abundance was assessed by quantifying the copy numbers of the ITS region using ITS1-F and 5.8S primers (5′-CGCTGCGTTCTTCATCG) (Vilgalys and Hester 1990). PCR mix contained 0.4 *μ*mol/L of each primer, 0.5× Power SYBR® Green, 1.0 mg/mL BSA, completed with nuclease-free water. Each PCR consisted of 23 *μ*L of PCR mix for fungi and 2 *μ*L of DNA template (100 ng/mL). The PCR run consisted of an initial denaturation step at 95°C for 10 min, followed by 40 cycles at 95°C for 60 s, 53°C for 30 s, and 72°C for 60 s.

Each run was run with a standard curve corresponding to serial dilutions of the *Escherichia coli*-derived vector plasmid JM 109 (Promega) containing either 16S rRNA gene (from *E. coli*) or ITS region (from *Rhizoctonia solani*). A six-point standard curve was carried out for each run for 10^8^–10^2^ molecules/*μ*L. The efficiency of the standard curve was calculated using the formula Eff = (10^(−1/slope)^−1) (102.3% for bacteria; 94.5% for fungi). We carried out the quantification in triplicate for standard curves as well as samples. Each run was ended with a dissociation stage (temperature set accordingly). The final abundance values were reported per 0.5 g of eggshell.

### Construction of 16S rRNA gene (bacteria) and ITS region (fungi) clone libraries

We constructed two bacterial clone libraries based on the 16S rRNA gene, and two fungal clone libraries based on the ITS region. PCR amplification of both the 16S rRNA gene and ITS region were performed following the same protocol described for bacterial and fungal PCR-DGGE, respectively, except for the forward primers of the nested PCR. Specifically, for both bacterial and fungal PCR, the forward primers were free of the GC-clamp, and the fungal forward primer (ITS1-F) was complete (5′-CTTGGTCATTTAGAGGAAGTAA) (Gardes and Bruns 1993).

For each set of early and late incubated eggs, we pooled ten nested PCR products together in order to identify bacterial and fungal communities. Each pool of nested PCR products was ligated into pGEM®-T-Easy vectors (Promega) and introduced into competent *E. coli* JM 109 cells according to the manufacturer's instructions (Promega). We tested about 20% of the white colonies to estimate cloning efficiency. For each library, 96 different colonies were picked and individually plated on LB agar completed with 100 *μ*g/mL ampicillin in a 96-well microtiterplate. Samples were sequenced by SeqLab (Göttingen, Germany).

### Sequence trimming and analysis of the clone libraries

Prior to sequence analyses, all obtained chromatograms were trimmed based on quality scores with an accuracy threshold of 0.2% using the algorithm LUCY (Chou and Holmes 2001), available within the Ribosomal Database Project pipeline (http://rdp.cme.msu.edu/). Vector sequences and sequences containing unascribed nucleotides or less than 300 bp for bacteria and 200 bp for fungi (in length) were also removed. The presence of chimeras was detected using Bellerophon v.3 on the Greengenes Web site (http://greengenes.lbl.gov).

Sequence analyses were performed by a phylogeny-based approach and applying operational taxonomic unit (OTU)-based analyses using the Mothur software package (Schloss et al. 2009). To generate richness and diversity estimators, and rarefaction curves, sequences were clustered at 99% and 98% nucleotide identity, for 16S rRNA and ITS data, respectively, using the default clustering method implemented in Mothur (i.e., Furthest neighbor algorithm). For 16S rRNA phylogenetic-based analyses, one representative sequence per OTU was used, as well as the best matched sequence per representative OTU sequence. Sequences were classified using the RDP taxonomy via RDP classifier (Wang et al. 2007). Sequences were aligned and visually inspected using MEGA v 4.0 (Tamura et al. 2007). The evolutionary history was inferred using the neighbor-joining method (Saitou and Nei 1987). The distances were computed using the Kimura-2 parameter method (Kimura 1980) and are in the units of the number of base substitutions per site (note scale bar – Fig 2). The branches were tested with bootstrap analyses (1000 replications), and trees were prepared for display using the online application “Interactive Tree Of Life” (iTOL) (http://itol.embl.de/) (Letunic and Bork 2007). For the ITS region, classification of the fungal sequences was carried out by comparing to those in the GenBank database, using the Basic Local Alignment Search Tool algorithm (BLAST) nt/nt (Altschul et al. 1997).

### Statistical analyses

The DNA concentrations, the average similarity of the community structure (only for *swab*/*crush* comparison because of pairwise data), the associated DGGE parameters (including number of bands, the Shannon index, and evenness values), and the log copy number of qPCR were all checked for normality and transformed when necessary. We assessed differences with Student's t-tests (two-tailed distribution). Statistical tests were based on the threshold *α *= 0.05 and considered significant when *P *< 0.05. We conducted the analyses using R 2.14.1 software (R Development Core Team, Vienna, Austria).

Our sample size varied between the different datasets: (i) we ran the comparison between eggshell pieces and egg swabs with ten eggs; (ii) the early/late incubation comparison consisted of twelve eggs each for bacterial community description; and (iii) we used ten eggs for early incubated eggs and increased this number up to sixteen for late incubated ones for fungal community description. The sampled eggs came from six different nests.

Analyses of DGGE profiles were performed using matrices based on band-matching surfaces. These matrices were used to calculate the Shannon index, evenness values, and to analyze community structure by exporting them into PRIMER v0.6 software (version 6; PRIMER-E Ltd, Plymouth, UK) (Clarke and Gorley 2006). Nonmetric multidimensional scaling (NMDS) graphs were generated from our dataset previously modified by a fourth-root transformation. Resemblance matrices were obtained using Bray–Curtis similarity (Euclidian distance gave similar results [data not shown]). We performed our statistical analyses based upon the analysis of similarity (ANOSIM; one-way analysis; 5000 permutations). The associated global R described the percentage of permutations related to the *P*-value, and the stress value indicated how faithful the relationships among samples are represented in the ordination plot.

## Results

### Microbial DNA extraction of eggs: egg swabs versus eggshell pieces

When comparing the methods based on swabbing, we found that the chelex protocol (Martín-Platero et al. 2010) gave inconsistencies with fluorescence readings, therefore impairing DNA quantification. Specifically, we observed high fluorescence values in the DNA-free control, sometimes higher than our sample values. We therefore discarded this method. Furthermore, DNA concentrations obtained from extractions with FSs kit were significantly higher than the ones obtained from both DNt and NSt kits (data not shown). On average, egg swabs had a DNA concentration of 264.6 ng/mL (±136.4; range: 12.0–1342.0), while eggshells had five times more DNA with 1517.3 ng/mL (±722.34; range: 21.0–6206.0; *t* = 3.18, df = 9, *P *= 0.01). Furthermore, when comparing the two kits used for DNA extraction using eggshell pieces, we found higher DNA concentrations and a higher number of bands per DGGE generated lane using the FSs kit instead of the NSs kit (data not shown). We concluded that FSs was the best performing kit for both egg swabs and eggshells and therefore used it to conduct our study.

When using DGGE profiles and associated band-matching surface values to compare egg swabs and eggshells, we found that DGGE lanes counted on average 16.8 (±1.51) bands for egg swabs and 15.6 (±1.17) bands for eggshells, a nonsignificant difference (*t* = 0.65, df = 9, *P *= 0.53). Both species diversity and evenness did not vary between extraction methods (*t* = 0.72, df = 9, *P *= 0.49, and *t* = 1.19, df = 9, *P *= 0.26, respectively). However, the resemblance matrix based upon DGGE lanes showed that swabs shared on average 51.9% (±2.44) similarity in their community structure, while eggshells shared significantly lower similarity among each other (43.4% ± 2.12; *t* = 2.64, df = 86, *P *= 0.009). Moreover, based on the same resemblance matrix, we calculated that swab/eggshell pairs shared on average 51.0% (±5.10) similarity (Appendices A1 and A2).

Regarding the 16S rRNA gene abundance, egg swabs had significantly fewer copy numbers (log 5.1 ± 0.18) than had eggshells (log 6.4 ± 0.48; *t* = 2.38, df = 9, *P *= 0.04; Appendix A1).

### Bacterial communities of early and late incubated pigeon eggs

**Figure 1:**
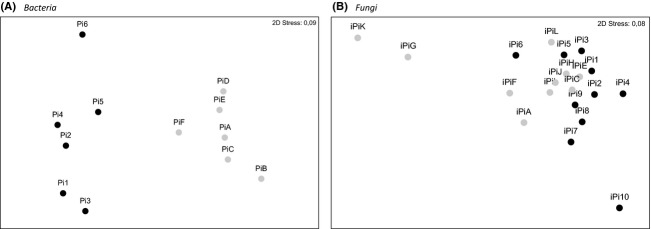
Nonmetric multidimensional scaling plots of bacterial and fungal communities on pigeon eggs based on denaturing gradient gel electrophoresis (DGGE) lane analysis. Early incubated eggs, annotated Pi*number* (A) or iPi*number* (B), are represent by *black dots*; late incubated eggs, annotated Pi*letter* (A) or iPi*letter* (B), are represent by *gray dots*. The nonmetric multidimensional scaling (NMDS) representation is based on a Bray–Curtis similarity matrix. A one-way analysis of similarity (ANOSIM) test determined that early and late incubated eggs were significantly different (*P *= 0.02, *R* = 0.92, stress = 0.09) for bacteria (A), but not significant for fungi (B) (*P *= 0.30, *R* = 0.028, stress = 0.08).

Denaturing gradient gel electrophoresis (DGGE) analysis, based on DNA extracted from eggshells, demonstrated that the number of bands at early incubation (18.5 ± 0.92) and late incubation (16.5 ± 0.50) did not significantly differ from each other (*t* = 1.91, df = 10, *P *= 0.09). Neither species diversity nor evenness differed between early and late incubated eggs (*t* = 1.34, df = 10, *P *= 0.21, and *t* = 0.86, df = 10, *P *= 0.41, respectively) (Appendix A3). However, based on the resemblance matrix generated from DGGE profiles, we noted a significant increase in the similarity from early to late incubated eggs (global *R* = 0.92; *P *= 0.002). Bacterial communities from early incubated eggs shared 65.8% (±2.39) similarity, while this similarity was of 75.5% (±2.11) at late incubation, regardless of nest of origin (Fig 1A; Appendix A3).

Bacterial abundance as measured by the log 16S rRNA gene copy number almost doubled over incubation time from log 3.6 (±0.32) for early incubated to log 6.3 (±0.18) for late incubated eggs, a significant increase (*t* = 7.42, df = 10, *P *< 0.001) (Appendix A3).

Clone library analysis was based upon the analysis of 92 sequences at early incubation and 95 at late incubation; sequences were grouped into 37 and 19 OTUs, respectively (Table 1), and the two libraries shared 7 OTUs. The estimated OTU richness (ACE and Chao1 indexes) was more than twice as high for early than for late incubated eggs. Similarly, the species diversity (Shannon index) was higher for early incubated eggs (Table 1). The lower bacterial diversity observed at late incubation implied higher sample coverage, confirmed by a stronger plateau in the rarefaction curve compared to the curve for early incubated eggs (Appendix A4).

**Table 1:** Effect of incubation on the diversity of bacterial and fungal communities associated with eggs.

	NS^1^	OTUs^2^	Estimated OTU richness		Shannon index	ESC^3^
			ACE	Chao1		
Bacteria						
Early incubation	92	37	167 (119; 244)	137 (69; 348)	3.16 (2.95; 3.38)	0.185
Late incubation	95	19	59 (39; 101)	64 (32; 167)	2.35 (2.16; 2.55)	0.406
Fungi						
Early incubation	65	15	15 (15; 22)	15 (15; 18)	2.47 (2.30; 2.64)	0.38
Late incubation	85	7	7 (7; 15)	7 (7; 7)	1.54 (1.38; 1.70)	0.53

OTU, operational taxonomic unit. Data are based on sequencing of the 16S rRNA gene (bacteria) and ITS region (fungi) obtained from two clone libraries: early and late incubated pigeon eggs. Given values correspond to their average (lowest; highest values).

1 Number of sequences for each clone library.

2 Calculated Mothur at 99% of nucleotide identity (16S) and at 98% of nucleotide identity (ITS).

3 Estimated sample coverage: *C*x = 1−(*N*x/*n*), where *N*x is the number of unique sequences, and *n* is the total number of sequences.

**Figure 2:**
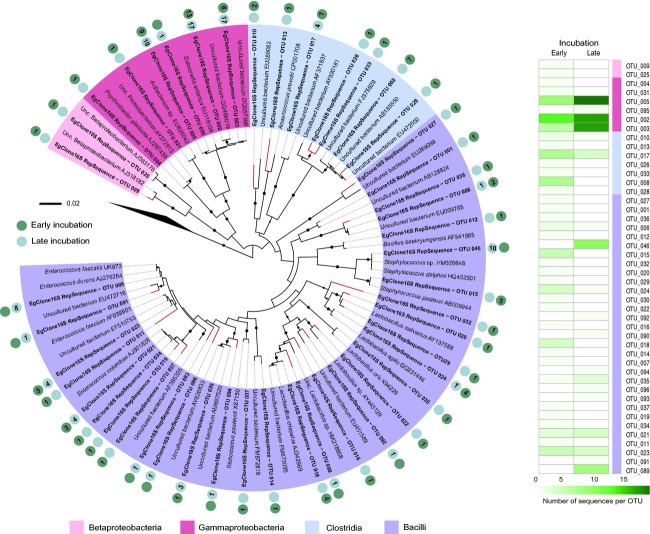
Phylogenetic analysis of bacterial 16S rRNA gene retrieved from early and late incubated eggs. Phylogenetic tree *–* Bootstrap values (1000 repetitions) above 50% are represented by solid circles next to tree branches. Eggshell derived clones are in bold, with their respective tree leaves marked in red. The tree displays one representative clone per operational taxonomic unit (OTU), and their respective best match sequence obtained from Ribosomal Database Project II. A single OTU embraces all clone sequences sharing at least 99% of nucleotide identity. Side circles next to eggshell clone labels indicate the number of clones belonging to the correspondent OTU at early (dark green) and late incubation (light green) side circles. Evolutionary relationships of 87 taxa encompassed the classes of *Betaproteobacteria* and *Gammaproteobacteria* within the phylum of *Proteobacteria*, and the classes of *Clostridia* and *Bacilli* within the phylum of *Firmicutes*. There were a total of 443 nucleotide positions in the final dataset and the phylum of *Aquificae* served as outgroup. Heatmap – Each representative clone per OTU from the phylogenetic tree is represented for early and late incubation. The intensity of the green is relative to the number of clone sequences embraced by this representative clone.

The phylogenetic classification of the bacterial sequences showed that early incubated eggs were mainly inhabited by *Firmicutes*, including 40.2% of *Bacilli* and 25.0% of *Clostridia-*affiliated species. *Proteobacteria* were also present and mainly represented by *Gammaproteobacteria* (32.6%) and to a smaller extent by *Betaproteobacteria* (2.2%). While the percentage of OTUs affiliated with *Bacilli* (39.0%) for late incubated eggs was close to early ones, only a few *Clostridia-*affiliated species remained present after incubation (4.2%). Additionally, we observed that *Gammaproteobacteria-*affiliated species were the only remaining representative of *Proteobacteria* at late incubation, and also the main one of the three bacterial classes, with 56.8% of the OTUs (Fig 2). In more detail, we observed that 28 clones from early incubated egg sequences were phylogenetically clustered with *Salmonella enterica* (*Gammaproteobacteria*). However, at late incubation, 54 clones were affiliated to this species. Similarly, while only five clones were closely related to *Staphylococcus sp*. (*Bacilli*) at early incubation, 10 clones were closely related to this genus at late incubation. Lastly, 12 clones from early incubated eggs and 19 clones from late incubated were affiliated with *Enterococcus sp*. (*Bacilli*) (Fig 2; Appendix A5).

### Fungal communities of early and late incubated pigeon eggs

PCR targeting fungi indicated their presence in all early incubated (*n *= 10) but only in half of the late incubated eggs (*n *= 10). Therefore, we increased our sample size to sixteen of which 10 (62.5%) contained fungal communities at late incubation.

Denaturing gradient gel electrophoresis (DGGE) analysis showed that the number of bands between early (7.2 ± 0.84) and late incubated eggs (8.7 ± 1.48) was similar (*t* = 0.88, df = 18, *P *= 0.39). Neither the species diversity nor the evenness showed differences during incubation (*t* = 0.35, df = 18, *P *= 0.73, and *t* = 1.14, df = 18, *P *= 0.27). Additionally, the similarity of these DGGE lanes remained comparable (Global *R* = 0.03; *P *= 0.30). At early incubation eggs shared 14.9% (±2.38) similarity, and 16.2% (±2.60) at late incubation (Fig 1B; Appendix A3).

DNA abundance changed during incubation. As mentioned previously, almost half of the eggshells did not have fungal DNA and had undetermined cycle threshold values at late incubation. Among the remaining late incubated eggs, we noticed a decrease in ITS region copy number from log 3.3 (±0.18) in early incubated eggs to log 2.6 (±0.18) at late incubation (*t* = 3.00; df = 18, *P *= 0.008) (Appendix A3).

Clone libraries led to the analysis of 65 sequences for early incubated and 85 sequences for late incubated eggs. Sequences were, respectively, grouped into 15 and 7 OTUs. Early and late incubated eggs had only three OTUs in common. Both ACE and Chao1 indexes of the estimated OTU richness were twice as high for early incubated eggs. Likewise, species diversity was higher for early incubated eggs (Table 1). Interestingly, the estimated sample coverage was higher for late incubated eggs, and its related rarefaction curve reached a well-marked threshold, meaning that almost the full fungal communities of our samples have been described (Table 1; Appendix A4).

**Figure 3:**
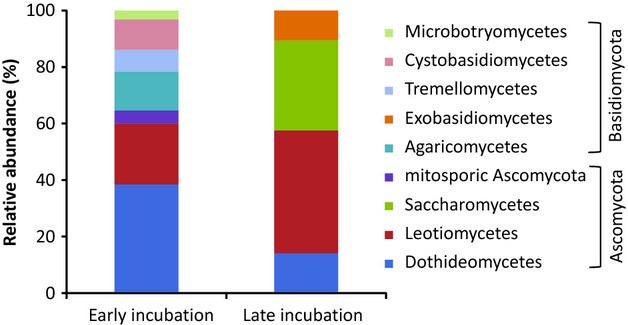
Relative abundance of fungal internal transcribed spacer (ITS) region derived operational taxonomic unit (OTUs) retrieved from early and late incubated eggs. Sequences were assigned to OTUs at 98% of nucleotide identity.

We classified fungal OTUs into *Ascomycota* and *Basidiomycota* phyla and further into classes. More than half of the OTUs from early incubated eggs were affiliated with *Ascomycota* (64.6%), mainly represented by *Dothideomycetes* (38.5%) and *Leotiomycetes* (21.5%), whereas the percentage of sequences affiliated with this phylum was even higher for late incubated eggs (89.4%). The sequences were spread over *Dothideomycetes* (14.1%), *Leotiomycetes* (43.5%), and *Saccharomycetes* (31.8%). For early incubated eggs, the *Basidiomycota* phylum contained *Agaricomycetes* (13.9%), *Tremellomycetes* (7.7%), *Cystobasidiomycetes* (10.8%), and *Microbotryomycetes* (3.1%). However, of the late incubated egg sequences, only 10.6% of the remaining OTUs belonged to *Basidiomycota*, and all corresponded to *Exobasidiomycetes*, which was not present at early incubation (Fig 3). In more details, when possible, each OTU was associated with a fungal species leading to their description at the genus or species level (Appendix A6).

## Discussion

Here, we made use of molecular tools to describe egg-related microbial communities. We first examined bacterial communities using two sampling techniques, egg swabs and crushed eggshells. Using the most adequate methodology (crushed egg shells), we compared the structure, abundance, and composition of both bacterial and fungal communities on early and late incubated eggs.

### Egg swabs versus eggshell pieces techniques to assess microbial communities in eggshells

The structure of bacterial communities based on eggshell pieces differed from those found using swabs, supporting similar findings from poultry sciences. In chicken eggs, it has been shown that depending on the method used to recover bacteria associated with the eggshell, different subsets of bacterial communities can be observed (Musgrove et al. 2005; Kawasaki et al. 2008; Chousalkar and Roberts 2012). The natural porosity of eggshells is likely the reason for these differences. For instance, aerobic bacteria recovered from “the crush method” exceeded the number of cells obtained using a “shell rinse method” (Kawasaki et al. 2008). In an experiment where eggshell surfaces were inoculated with *Salmonella* cells, the number of cells obtained from crushed eggshells previously washed was about half the number of cells recovered from their own egg rinsates (Kawasaki et al. 2008). In line with these studies, we detected more than five times the amount of microbial DNA when using the whole eggshell, enabling a proper quantification of the DNA concentration in each sample through fluorescence. The DNA concentration was sufficiently large to perform a range of molecular analyses to determine abundance and diversity of bacterial and fungal communities associated with eggshell in the same sample.

Importantly, our results showed that different extraction methods uncover different bacterial communities, as the overall bacterial structure based on eggshell swabs exhibited low levels of similarity when compared to the eggshell pieces coming from the same egg. Moreover, when comparing different eggs, bacterial communities obtained by the swab method shared higher similarity than did those obtained by crush method (eggshell pieces). In addition, for comparative purposes, we had to set the template DNA used for PCR-DGGE at the same concentration for both methods. Because the DNA concentration obtained with the swab method was low and suboptimal when compared to other studies dealing with environmental samples (Pereira e Silva et al. 2012), this limited our bacterial analyses for comparison. The use of the eggshell pieces allowed us to overcome the limitations of low-template concentration and therefore provided a more complete description of community structure and diversity of microorganisms associated with the eggshells.

Despite the advantages that using eggshell pieces provide for the molecular analyses of microbial communities, egg destruction might not be desirable in every study. In particular, the use of eggshell swabs may remain unavoidable in studies dealing with embryo survival (Martín-Gálvez et al. 2010). Our results raise awareness that swabbing does not capture the entire microbiome, and therefore, the findings obtained based on this method should be interpreted keeping this limitation in mind. For studies that focus on egg contents (and require egg destruction), we advocate that working with eggshells is a better alternative.

### Shifts in microbial communities through incubation

It has been hypothesized that incubation reduces the water on the egg surface to prevent bacterial growth (Cook et al. 2003, 2005a,b2005b; D'Alba et al. 2010; but see Wang et al. 2011), when compared to exposed (unincubated) eggs. This bacteriostatic effect of incubation has been attributed as an important mechanism controlling the hatching success (Cook et al. 2003, 2005a; Beissinger et al. 2005). It is less clear, however, if microbial communities change during incubation, and if this can be correlated with embryo survival. Even though the presence of bacteria is often seen as a potential risk for embryo development, due to the presence of pathogenic species, it is important to realize that bacterial communities are highly diverse and that only a small subset of the total comprise pathogens. Additionally, the concept of pathogenicity cannot be generalized to a certain genus or species, as it depends on an intricate interaction between bacterial strain, host species, and host health. Alternatively, the microbial communities associated with eggshells can be seen as a protective barrier against pathogenic species. It has been hypothesized that incubation should prevent the growth of pathogenic strains while enhancing the growth of beneficial ones (Cook et al. 2005b; Shawkey et al. 2009).

Considering the bacterial communities, our results showed an overall decrease in diversity during incubation, whereas a few bacterial OTUs that preferentially grew to the detriment of others, remained after 3 weeks of incubation. The higher number of specific bacterial OTUs observed at late incubation might explain the overall increase in bacterial abundance. One possible explanation is that bacteria that are selected during incubation probably grow in the empty places left by dead cells as is the case in bacterial soil invasion (Eisenhauer et al. 2013). Our results contrast with the only molecular study addressing this issue, on Pearly-eyed Thrashers (Shawkey et al. 2009), where bacterial abundance and structure did not change during incubation. However, this disparity could be explained by methodological issues. First, Shawkey et al. (2009) used the swab method for DNA extraction, which does not comprise the bacterial communities found inside the eggshell pores. Second, Shawkey et al. (2009) quantified bacterial abundance based on the overall DNA concentration obtained per sample, which includes other sources of DNA (fungal, animal) in addition to that of bacterial origin. In our case, we used more specific methods, such as qPCR, which is commonly used to quantify microbial DNA in a range of environmental samples (Bach et al. 2002; Pereira e Silva et al. 2012). Third, the contrasting results might be a reflection of the environment where the samples were collected and bird species. Moreover, our study was performed on captive birds in semi-natural conditions, on unfertilized eggs, and may thus not be entirely representative of what happens in the wild. Further molecular studies are needed to unravel how bacterial communities vary through the incubation period.

Here, we reported that late incubation favoured mostly species belonging to the *Gammaproteobacteria* class, which comprised 57% of the total number of OTUs found, whereas this bacterial class accounted for 22% on early incubated eggs. These changes were mostly due to the presence of OTUs affiliated to *Salmonella enterica* (range: 98–99% similarity, when the closest hit was assigned at the genus level, Appendix A5), which belongs to the family Enterobacteriaceae. Culture-dependent and molecular methods have detected this bacterial family at both early and late incubation stages (Shawkey et al. 2009; Ruiz-de-Castañeda et al. 2011b), indicating that this family is an important component of the egg microbiome. Based on molecular methods, Shawkey et al. (2009) have shown that the number of bacterial taxa belonging to the Enterobacteriaceae family tended to increase from early to late incubation, even though a significant increase was observed only when comparing unincubated and incubated eggs (Shawkey et al. 2009). We also observed a twofold increase in *Staphylococcus-*like OTUs throughout incubation (order *Bacillales*). Thus, although incubation might limit the growth of potential pathogenic bacterial species, our study shows that some of these species tend to increase from early to late incubation. However, their actual role remains unclear due to the lack of information on their pathogenicity. Testing for bacterial pathogenicity would require *in vivo* experiments (when the specific isolates are available) or the use of more specific molecular tools targeting type III and/or IV secretion systems (see Deane et al. 2010) for instance. To our knowledge, only one study established a real effect of bacteria on bird fitness: Soler et al. (2012) have shown that Enterococcus and Enterobacteriaceae on eggshell were negatively associated with hatching success. Moreover, while pathogenicity is often argued when Enterococcaceae, Enterobacteriaceae, or Staphylococcaceae are described, none of the studies discussed their potential beneficial or commensal role. For instance, in humans, some of these family-related species were often found in the gut without being consistently harmful (i.e., Enterococci described in Byappanahalli et al. 2012). Considering that pathogenicity is most likely strain-and host-dependent, additional in-depth studies are certainly required for more conclusive explanations.

Fungi are undoubtedly part of the microbiome associated with the egg environment. They have been described in nest materials and might colonize eggshells (Baggott and Graeme-Cook 2002). It has been postulated that fungi might be able to break down the cuticle to facilitate bacterial trans-shell penetration, by increasing the number of pores accessible (Board et al. 1964, 1979; Board and Halls 1973). The probability of bacterial infection of egg contents was shown to be positively associated with fungal growth on eggshells (Cook et al. 2003). Moreover, fungi have been described in poultry industries (see Szablewski et al. 2010; Nowaczewski et al. 2011). Nevertheless, comparing our results with previous studies proved to be challenging because of the lack of data on fungi associated with eggshells. So far, they have been detected in egg contents after exposure to their natural environmental conditions (Cook et al. 2003, 2005a). Using cultivation methods, Godard et al. (2007) showed that eggshells were free from fungi most of the time at the laying day, but their number increased over time on exposed eggs, possibly due to the water on the egg surface. Conversely, using molecular tools, we showed that fungi are a constant constituent of the egg microbiome at early incubation, although their importance reduced during incubation, as only about half of the eggshells still harbored fungal DNA at the late incubation period. This reduction could be due to the humidity control of the egg caused by incubation behavior, because fungi abundance can be correlated with the water content on the eggshell (Godard et al. 2007). Additionally, we observed a strong decrease in their diversity, indicating that only a few selected species were able to cope with the lower level of humidity. However, as we did not measure water content on the eggshell, we cannot discriminate between resistance to low humidity or differences in humidity between eggs.

Interestingly, some of the fungi identified in our study have been reported in the avian gut microbiota, and their prevalence may depend on the avian species (Cafarchia et al. 2006). This includes *Cryptococcus laurentii*,*Cryptococcus uniguttulatus*,*Debaryomyces hansenii,* and *Rhodotorula rubra,* which have been identified in feral pigeon cloaca (Littman and Walter 1967; Mattsson et al. 1999). Up to fourteen fungal species have been also described in the lower intestinal tract of these birds (Ramirez et al. 1976). The presence of fungi on the eggshells of early incubated eggs might be explained under the hypothesis of vertical transmission. Additionally, some fungi may grow through incubation while others die out. The presence of new OTUs after incubation could be explained by other sources as fungi have been for instance found in feathers of pigeons (Deshmukh 2004) and other avian species (Mandeel et al. 2011) and often mentioned in avian ecological studies (i.e., Bisson et al. 2007; Brilhante et al. 2012). More molecular-based studies focusing on other bird species are needed to determine whether these results are consistent or not between bird species and environmental conditions, and the possible role of fungal species on embryo survival.

## Further Perspectives

This paper provides a description of bacterial and fungal communities on avian eggshells. Our results showed for the first time that bacterial growth increases during incubation because this behavior led to an increase in the overall bacterial abundance, which could be linked to the dominance of specific bacterial types, as shown by a reduction in diversity. However, opposite results were found for fungi, whose abundance and diversity decreased through incubation. The evolutionary and ecological roles of fungi on eggshells are still unclear, and further investigations are needed to understand the consequences of microbial communities on bird fitness. Investigating eggs from different species with different shell structure may lead to different microbial communities (Massaro et al. 2004; Zimmermann and Hipfner 2007). Consequently, investigating other avian species might reveal if and when incubation behavior selects specific microbial species in order to ensure embryonic survival. In this context, the use of eggshells might represent a valuable option in studies trying to relate microbial infection and antimicrobial defences (Horrocks et al. 2012).

## Figures and Tables

**Figure 4:**
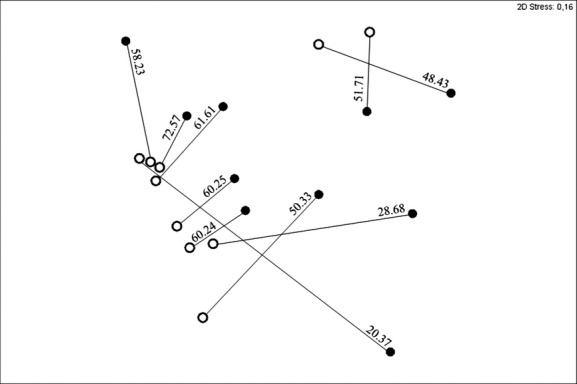
Nonmetric multidimensional scaling plot of bacterial communities per pair of pigeon eggshells. For each pair, bacterial communities were obtained either by crush method (full circles) or by swabbing method (open circles) of the eggshell. Each dot on the graph represents its relative position based on DGGE band-matching surface. The similarity within pairs is given by a similarity value (in percentage) on the top ofeach line, based on Bray–Curtis similarity matrix.

**Figure A4:**
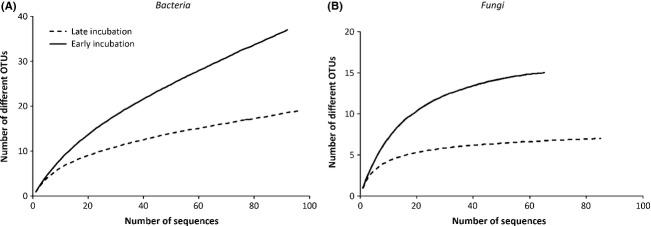
Rarefaction curves of operational taxonomic units (OTUs) of bacterial 16S rRNA gene and fungal ITS region. Sequences were obtained from two clone libraries: early *(solid line)* and late incubated *(dotted line)* eggs. OTUs were clustered at 99% of nucleotide identity for bacteria (A) and at 98% identity for fungi (B).

## Data Accessibility

DNA sequences: bacterial 16Sr RNA gene: GenBank accessions KF932474–KF932565 (early incubation), accessions KF932379–KF932473 (late incubation). Fungal ITS-1 region: GenBank accessions KF932566–KF932630 (early incubation), accessions KF932631–KF932715 (late incubation).

## Appendix A1

**Table A1:** Effect of sampling technique (egg swabs vs. eggshell pieces) on bacterial diversity and abundance.

	Band counting	Shannon index	Pielou's index	Similarity (%)	Gene copy (log)
Egg swabs	16.8 (±1.51; 10–23)	2.8 (±0.10; 2.6–3.1)	1.0 (± <0.001)	51.9 (±2.44; 20.2–83.0)	5.1 (±0.18; 4.0–5.7)
Egg swabs/eggshell pieces^1^	–	–	–	51.0 (±5.10; 20.4–72.6)	–
Eggshell pieces	15.6 (±1.17; 10–21)	2.8 (±0.10; 2.3–3.0)	1.0 (± <0.001)	43.4 (±2.12; 22.1–73.7)	6.4 (±0.48; 3.6–8.9)

Band counting numbers, Shannon index (diversity), Pielou's index (evenness), and similarity are based on DGGE band-matching surface, and gene copy (log) (abundance) based on qPCR. Results are presented with their average (± standard deviation; minimal–maximal values).

1 For the comparison egg swabs versus eggshell pieces, only the value of similarity is given. The analysis was run on paired data.

## Appendix A3

**Table A3:** Structure and abundance of microbial communities associated with eggshells at early and late incubation.

	Band counting	Shannon index	Pielou's index	Similarity (%)	Gene copy (log)
Bacteria					
Early incubation	18.5 (±0.92; 15–21)	2.8 (±0.06; 2.6–3.0)	1.0 (± <0.001)	65.8 (±2.39; 51.0–80.9)	3.6 (±0.32; 2.9–5.0)
Late incubation	16.5 (±0.5; 15–18)	2.74 (±0.02; 2.7–2.8)	1.0 (± <0.001)	75.5 (±2.11; 62.1–93.8)	6.3 (±0.18; 5.4–6.7)
Fungi					
Early incubation	7.2 (±0.84; 4–11)	1.9 (±0.12; 1.3–2.4)	1.0 (± <0.001)	14.9 (±2.38; 0.0–55.1)	3.3 (±0.18; 2.6–4.5)
Late incubation	8.7 (±1.48; 2–16)	2.0 (±0.22; 0.7–2.7)	1.0 (± <0.001)	16.2 (±2.60; 0.0–54.3)	2.6 (±0.18; 2.0–3.5)

Band counting numbers, Shannon index (diversity), Pielou's index (evenness), and similarity are based on DGGE band-matching surface, and gene copy (log) (abundance) based on qPCR. Results are presented with their average (± standard deviation; minimal–maximal values).

## Appendix A5

**Table A5:** Identification of the bacterial OTUs retrieved from early and late incubated eggs.

	Affiliated sequences (%)	Affiliated sequences (%)	Affiliation^1^				
OTU identity	*Early incubation*	*Late incubation*	Phylum	Class	Closest hit^2^,^3^	Accession number	Similarity (%)^4^
001^5^	1.1	–	Firmicutes	Bacilli	*Bacillus azotoformans*	AB363732	85
002^5^	14.1	17.9	Proteobacteria	Gammaproteobacteria	*Salmonella enterica*	HF969015	99
003^5^	6.5	17.9	Proteobacteria	Gammaproteobacteria	*Salmonella enterica*	CP005995	99
004	1.1	–	Proteobacteria	Gammaproteobacteria	*Psychrobacter glacinola*	AJ297439	99
005^5^	9.8	20.0	Proteobacteria	Gammaproteobacteria	*Salmonella enterica*	CP005995	99
006^5^	2.2	1.1	Firmicutes	Bacilli	*Bacillus ginsenggisoli*	AB245379	68
007	1.1	–	Firmicutes	Bacilli	*Trichococcus pasteurii*	X87150	97
008^5^	7.6	–	Firmicutes	Clostridia	*Clostridium* sp.	AB596881	100
009^5^	1.1	–	Proteobacteria	Betaproteobacteria	*Burkholderia nodosa*	AY533861	94
010^5^	2.2	–	Firmicutes	Clostridia	*Parvimonas micra*	JN713239	66
011	1.1	–	Firmicutes	Bacilli	*Enterococcus faecium*	AF039901	90
012	1.1	–	Firmicutes	Bacilli	*Bacillus baekryungensis*	AF541965	99
013	1.1	–	Firmicutes	Clostridia	*Anaerococcus prevotii*	CP001708	71
014^5^	1.1	–	Firmicutes	Bacilli	*Alloiococcus otitis*	AB680896	85
015	5.4	–	Firmicutes	Bacilli	*Staphylococcus pasteuri*	AB009944	100
016^5^	1.1	–	Firmicutes	Bacilli	*Lactobacillus crispatus*	AB425941	88
017^5^	7.6	4.2	Firmicutes	Clostridia	*Clostridium* sp.	FJ384373	100
018	4.4	–	Firmicutes	Bacilli	*Lactobacillus crispatus*	AJ242969	99
019	1.1	–	Firmicutes	Bacilli	*Enterococcus columbae*	AJ301828	94
020	1.1	–	Firmicutes	Bacilli	*Lactobacillus salivarius*	AY137588	94
021	3.3	4.2	Firmicutes	Bacilli	*Enterococcus columbae*	AJ301828	100
022	1.1	–	Firmicutes	Bacilli	*Lactobacillus* sp.	AY445129	89
023	5.4	4.2	Firmicutes	Bacilli	*Enterococcus faecium*	AF039901	99
024	4.3	1.1	Firmicutes	Bacilli	*Lactobacillus agilis*	GQ231446	99
025^5^	1.1	–	Proteobacteria	Betaproteobacteria	*Ralstonia* sp.	AY509958	97
026^5^	1.1	–	Firmicutes	Clostridia	*Clostridium* sp.	JQ248565	90
027^5^	1.1	–	Firmicutes	Bacilli	*Trichococcus pasteurii*	L76599	96
028^5^	1.1	–	Firmicutes	Clostridia	*Clostridium* sp.	AB596881	94
029	1.1	–	Firmicutes	Bacilli	*Lactobacillus salivarius*	AY137588	83
030	1.1	–	Firmicutes	Bacilli	*Lactobacillus oris*	X94229	99
031	1.1	–	Proteobacteria	Gammaproteobacteria	*Avibacterium* sp.	EU826042	99
032	1.1	–	Firmicutes	Bacilli	*Lactobacillus agilis*	GQ231446	86
033^5^	1.1	–	Firmicutes	Clostridia	*Clostridium* sp.	EU869235	80
034	1.1	–	Firmicutes	Bacilli	*Enterococcus columbae*	AJ301828	94
035^5^	1.1	3.2	Firmicutes	Bacilli	*Aerococcus viridans*	JF496382	100
036^5^	1.1	–	Firmicutes	Bacilli	*Bacillus* sp.	FJ348039	68
037	1.1	–	Firmicutes	Bacilli	*Enterococcus columbae*	AJ301828	91
046	–	10.5	Firmicutes	Bacilli	*Staphylococcus* sp.	HM028646	100
089	–	8.4	Firmicutes	Bacilli	*Enterococcus durans*	AJ276354	99
090^5^	–	1.1	Firmicutes	Bacilli	*Lactobacillus gallinarum*	AB008208	85
091	–	1.1	Firmicutes	Bacilli	*Enterococcus durans*	AJ276354	92
092^5^	–	1.1	Firmicutes	Bacilli	*Lactobacillus* sp.	EF187258	89
093^5^	–	1.1	Firmicutes	Bacilli	*Enterococcus asini*	GQ337016	87
094^5^	–	1.1	Firmicutes	Bacilli	*Aerococcus viridians*	JF496443	86
095^5^	–	1.1	Proteobacteria	Gammaproteobacteria	*Salmonella enterica*	JQ694378	98
096	–	1.1	Firmicutes	Bacilli	*Enterococcus faecalis*	UK873	86

1 The taxonomic affiliation is presented at the phyla, class, and genus/species levels and was based on a single representative sequence from each operational taxonomic unit (OTU) clustered at 99% of nucleotide identity.

2 The phylogenetic classification was based on a single representative sequence from each OTU clustered at 99% of nucleotide identity.

3 When an uncultured bacterium was the closest hit to our sequences, it is the closest genus hit which is mentioned.

4 The representative sequence was compared with RDP database allowing establishing similarity shared (in percentage) with a reference sequence.

5 Uncultured bacterium was the closest hit.

## Appendix A6

**Table A6:** Identification of the fungal OTUs retrieved from early and late incubated eggs.

			Affiliation^1^(%)				
OTU identity	Affiliated sequences (%)	*Sample origin*	Phylum	Class	Closest hit^2^	Accession number	Similarity (%)^3^
1	12.3	Early incubation	Ascomycota	Dothideomycetes	*Cladosporium cladosporioides*	JN650537	100
2	1.5	Early incubation	Basidiomycota	Microbotryomycetes	*Rhodotorula dairenensis*	JN246550	100
3	3.1	Early incubation	Ascomycota	Dothideomycetes	*Cladosporium langeronii*	HQ115727	100
4	12.3	Early incubation	Ascomycota	Leotiomycetes	Uncultured Helotiales	JQ991733	96
5	16.9	Early incubation	Ascomycota	Dothideomycetes	*Davidiella tassiana*	AM159622	100
6	7.7	Early incubation	Basidiomycota	Cystobasidiomycetes	*Sporobolomyces foliicola*	AF444521	97
7	10.8	Early incubation	Basidiomycota	Agaricomycetes	*Psilocybe* spp.	DQ002870	100
8	9.2	Early incubation	Ascomycota	Leotiomycetes	*Sclerotinia sclerotiorum*	AB693927	100
9	3.1	Early incubation	Ascomycota	Dothideomycetes	*Phoma herbarum*	GU004222	100
10	3.1	Early incubation	Basidiomycota	Cystobasidiomycetes	*Sporobolomyces coprosmae*	AM160645	100
11	3.1	Early incubation	Ascomycota	Dothideomycetes	*Phoma* spp.	FJ430739	99
12	7.7	Early incubation	Basidiomycota	Tremellomycetes	*Cryptococcus* spp.	AM160648	100
13	1.5	Early incubation	Basidiomycota	Microbotryomycetes	*Sporobolomyces lactosus*	AB038132	99
14	4.6	Early incubation	Ascomycota	Leotiomycetes	*Tetracladium marchalianum*	FJ205463	99
15	3.1	Early incubation	Basidiomycota	Agaricomycetes	*Bjerkandera adusta*	JF340266	99
1	1.2	Late incubation	Ascomycota	Dothideomycetes	*Cladosporium cladosporioides*	JN650537	100
5	12.9	Late incubation	Ascomycota	Dothideomycetes	*Davidiella tassiana*	AM159622	100
8	5.9	Late incubation	Ascomycota	Leotiomycetes	*Sclerotinia sclerotiorum*	AB693927	100
16	35.3	Late incubation	Ascomycota	Leotiomycetes	*Lachnum* sp.	AB481283	93
17	31.8	Late incubation	Ascomycota	Saccharomycetes	*Debaryomyces hansenii*	HE660057	100
18	2.4	Late incubation	Ascomycota	Leotiomycetes	*Blumeria graminis*	AB273567	99
19	10.6	Late incubation	Basidiomycota	Exobasidiomycetes	*Malassezia globosa*	AY743630	97

1 The taxonomic affiliation is presented at the phyla, class, and genus/species levels and was based on a single representative sequence from each operational taxonomic unit (OTU) clustered at 98% of nucleotide identity.

2 The phylogenetic classification was based on a single representative sequence from each OTU clustered at 98% of nucleotide identity.

3 The representative sequence was compared with fungal database, allowing establishing similarity shared (in percentage) with a reference sequence.
